# Efficacy and safety of pharmacogenomic-guided antidepressant prescribing in patients with depression: an umbrella review and updated meta-analysis

**DOI:** 10.3389/fpsyt.2024.1276410

**Published:** 2024-07-11

**Authors:** Kiflu G. Tesfamicael, Lijun Zhao, Rubén Fernández-Rodríguez, David L. Adelson, Michael Musker, Thomas M. Polasek, Martin David Lewis

**Affiliations:** ^1^ School of Biological Sciences, University of Adelaide, Adelaide, SA, Australia; ^2^ Lifelong Health, South Australian Health and Medical Research Institute (SAHMRI), Adelaide, SA, Australia; ^3^ Adelaide Medical School, University of Adelaide, Adelaide, SA, Australia; ^4^ Health and Social Research Center, Universidad de Castilla-La Mancha, Cuenca, Spain; ^5^ Adelaide Nursing School, Faculty of Health and Medical Sciences, University of Adelaide, Adelaide, South Australia, Australia; ^6^ Centre for Medicine Use and Safety, Monash University, Melbourne, VIC, Australia

**Keywords:** depression, antidepressant, pharmacogenomics (PGx), pharmacogenomics-guided prescribing, genotyping panels

## Abstract

**Aim:**

To determine the efficacy and safety of pharmacogenomics (PGx)-guided antidepressant prescribing in patients with depression through an umbrella review and updated meta-analysis.

**Methods:**

A comprehensive systematic search was conducted on PsycINFO, PubMed, Embase and the Cochrane databases. The pooled effect sizes of randomized controlled trials (RCTs) were expressed as mean differences for continuous data and risk ratios for noncontinuous data.

**Results:**

Patients who received PGx-guided medications were 41% to 78% more likely to achieve remission and 20% to 49% more likely to respond to antidepressants than patients receiving treatment-as-usual (TAU).

**Conclusion:**

PGx-guided antidepressant prescribing improves the treatment of depression. However, the significance and magnitude of the benefit varies widely between studies and different PGx testing panels.

**Systematic review registration:**

https://www.crd.york.ac.uk/prospero/, identifier CRD42022321324.

## Introduction

Depression is one of the largest contributors to disability and suicide, with around 800,000 suicides per year worldwide (1). The prevalence of depression has increased by more than 25% over ten years (2005 - 2015) (2, 3). This increase is associated with a socioeconomic burden that costs billions of dollars annually (4). Moreover, the COVID-19 pandemic increased cases further, with an estimated global prevalence of 28% (2). Pharmacotherapy is the first-line treatment for moderate-to-severe depression (5). However, a significant proportion of patients fail to respond to medication (6). Up to 60% of patients with depression do not respond to their initial treatment and often switch from the first prescribed medication to other alternatives (6, 7). The likelihood of patients having a clinically positive response substantially decreases with subsequent treatments (6). The same antidepressant can be efficient in some individuals/populations but inefficient or may precipitate adverse drug reactions (ADR) in others (8). Therefore, new strategies are focused on personalizing the prescribing of antidepressant medications. This is part of widespread efforts across clinical practice to improve patient outcomes using precision medicine technologies, including precision dosing (9, 10).

Using an individual’s genotype to aid drug and dose selection, referred to as pharmacogenomics, is a promising approach with the potential to improve the treatment of depression (9–13). The field was initially called pharmacogenetics because it involved single genes or a combination of a relatively few number of genes, but this evolved into pharmacogenomics (PGx) to accommodate the many genes across the genome that influence gene-drug interactions (13). There are many commercial PGx testing panels available, including GeneSight, NeuroIDgenetix, CNSDose, Neuropharmagen and Genecept (12). Some panels offer clinical interpretation and decision-support tools related to psychiatric medications in addition to providing PGx testing (14, 15). Doctors may request PGx testing for patients either proactively to guide new medication prescribing or reactively if treatment fails.

In addition, there are regulatory bodies (US Food and Drug Administration, FDA) and research consortia (Clinical Pharmacogenetics Implementation Consortium, CPIC) that provide recommendations and guidelines for prescribing (12, 14–16). Among non-cancer drugs, psychiatric drugs have the highest proportion of drugs with FDA-approved PGx information (17). The FDA labelled 38 psychiatric drugs with PGx precautions, which are primarily metabolized by two major liver enzymes, CYP2D6 and CYP2C19, encoded by the highly polymorphic *CYP2D6* and *CYP2C19* genes, respectively (18). However, the clinical use of PGx testing in psychiatry is still low (19) and is challenging for many reasons, including CYP enzyme phenoconversion, poor adherence to medication regimens, negative lifestyle influences (e.g., tobacco smoking), and limited prescriber knowledge (20).

Numerous clinical trials, meta-analyses and systematic reviews have examined the efficacy and safety of PGx-guided drug selection for treating depression. Most of these studies have found that PGx-guided antidepressant prescriptions are superior to treatment-as-usual (prescribing without considering PGx test results (21–24). For example, Han et al. (24), reported that PGx-guide treatment led to a 50% or more reduction in depression severity than treatment-as-usual (TAU). These studies have demonstrated the importance of PGx testing in treating depression and have recommended its integration into standard care for depressed patients. On the other hand, several other studies reported a negative result or no impact of PGx-guided antidepressant selection (25, 26). These conflicting findings pose a major challenge for integrating PGx testing into clinical practices in psychiatry (27).

Therefore, it is imperative to thoroughly review the available evidence to summarize the findings in this area and support clinical practice guidelines. In this study, we conducted an umbrella review and updated meta-analysis to provide an overview of the safety and efficacy of using PGx testing to guide the selection of antidepressants in patients with depression as compared to TAU. We analyzed and summarized existing evidence from systematic reviews, meta-analyses and primary studies to present a compiled study on the impact of PGx-guided antidepressant prescription in treating depression. This document can be a helpful resource for clinicians and policymakers to understand the efficacy and safety of PGx-guided antidepressant treatment and make informed decisions. For the updated meta-analysis, we considered studies published after 2020 to provide the most recent evidence in the field and to investigate the influence of recent technological advancements in PGx testing on its effectiveness. This is important to examine how technological advancements in PGx testing technologies impacted the efficiency of PGx testing.

## Methods

This umbrella review of systematic reviews and meta-analyses and updated meta-analysis were conducted based on the standardized criteria according to the Preferred Reporting Items for Systematic Reviews and Meta-analysis (PRISMA) (28) and followed the JBI methodology for randomized controlled trials (RCTs) and systematic reviews of effectiveness (29). This review is registered with PROSPERO (CRD42022321324). It is important to note that the initial aim of the review was to conduct a systematic review; however, during the search process, sufficient meta-analysis and systematic review studies were discovered, which led to the decision to conduct an umbrella review.

### Search strategy and study selection

The authors conducted a three-step strategy searching for published studies on the impact of PGx-guided medications in treating depression. Initially, a limited search was conducted on PubMed and Google Scholar databases to identify search terms of relevance. The syntax of index terms was amended and used to develop a logic grid for each included database (**Supplementary Tables S6**, **S7**). After that, a comprehensive search was conducted on PsycINFO, PubMed, Embase and the Cochrane database from inception up to September 16^th^ 2022. ^A^ separate search was conducted for systematic reviews and RCTs. For RCTs, the search was limited from 2020 in order to provide updated evidence and to examine the potential impact of recent technological advancements in PGx testings on the efficiency of PGx-guided drug selection. Finally, additional articles not identified in the primary search were retrieved from a manual search of the reference lists of all included studies. The search was restricted to studies written in English.

All identified studies were collated and uploaded into EndNote 20 (Clarivate Analytics, PA, USA) and duplicated articles were removed. Then, all studies were uploaded into Covidence (30) which supported removing the remaining duplicates and conduct screening, quality assessment and data extraction. First, studies were screened based on their title and abstract and eligible studies were retrieved for full-text assessment. Two independent reviewers (KT & LZ) screened all identified articles against the eligibility criteria according to the following PICOs strategy: i) Population: adults (aged ≥18 years) with depression diagnosed by clinicians; ii) Intervention: utilized PGx-guided medications; iii) Comparator: standard treatment or TAU; iv) Outcomes: related to the efficacy and safety of the intervention (i.e., symptom improvement, rate of response, rate of remission, drug tolerance, side effects and other safety outcomes); v). Studies: systematic reviews, meta-analyses, blinded RCTs and controlled open-label design studies published in peer-reviewed journals. Disagreement between reviewers at all levels was discussed until a consensus was reached, and arbitration was completed with a third reviewer (MM) if an agreement was not reached. We excluded association or cost-effectiveness studies, studies with missing data, posters or conference papers.

### Assessment of risk of bias and small studies effect

Two independent reviewers (KT & LZ) critically appraised all articles selected for inclusion at the study level for methodological validity and the risk of bias in their design, research conduct and analysis. The critical appraisal was completed using JBI-standardized critical appraisal tools for RCTs and systematic reviews (31). Corresponding authors were contacted for missing information or when additional clarification was required. Critical appraisal results were presented in a table and summarized. Based on the score, the included systematic reviews and meta-analysis studies were categorized as very low (0-3), low (4-6), moderate (7-9), and high (10-11) scores. All studies, irrespective of methodological quality, underwent data extraction and synthesis. The limitations and methodological quality were reported and discussed.

### Data extraction

Two independent reviewers (KT and LZ) extracted the following data from systematic reviews and meta-analysis with a customized template on Covidence (30): author and year of publication, type of review, number of participants in the intervention and control groups, number of included studies, participant characteristics, outcomes of interest, a summary of findings, effect sizes with 95% confidence intervals (CIs) and the heterogeneity of the meta-analysis (if conducted) indicated by the *I^2^* statistic. For RCT, we extracted the author and year of publication, study design, length of the intervention, number of participants and events in each group, outcomes of interest, and the list of variants genotyped and utilized the PGx testing panel. When information was missing or incomplete, we contacted the corresponding authors. When authors failed to provide the missing data, we extracted the standard error (SE) or standard deviation (SD) data from graphs or figures using the WebPlotDigitizer (32).

### Statistical analysis

#### Primary analysis

For the umbrella review, we designed forest plots to visually display and synthesize the pooled effect size estimates with their corresponding 95% CIs and heterogeneity value (*I* ^2^) for the rate of remission and rate of response from the included meta-analysis. Results from the systematic review studies were narrated and summarized.

#### Updated meta-analysis

An updated meta-analysis was conducted to measure symptom improvement, rate of remission, and rate of response to PGx vs TAU. We extracted the percentage of mean changes in depression score from baseline to post-intervention and the number of individuals that achieved the predefined thresholds for rate of response and rate of remission. The SD of each mean percentage change was extracted and used for the analysis. Subsequently, we pooled the effect sizes from each study and for each outcome. Effect sizes were expressed as final post-intervention mean percentage differences for continuous outcomes (symptom improvement) or risk ratios (RR) for categorical outcomes (remission and response rate). Data were analyzed using Review Manager (RevMan) version V5.4 (33). The RR for each study was calculated by comparing the counts (events) of response and remission within PGx-guided and TAU groups and pooled using a random-effect model using the inverse variance method (34). The random-effect model was used to address the study design difference (open-label and RCTs) across the included studies. A subgroup analysis was performed to determine the potential influence of the PGx tests utilized on the effect sizes. Furthermore, sensitivity analyses were conducted for each outcome to determine the robustness of the summary estimates by removing one-by-one each included RCT. Heterogeneity of the effect sizes across selected studies was tested using standard *Chi* ^2^ with *P* < 0.10, indicating significant heterogeneity and its magnitude was measured using the *I* ^2^ statistic. *I* ^2^ values of less than 40%, 30% - 60%, 50% - 90% and 75% - 100% considered may be low, moderate, substantial and considerable heterogeneity, respectively (35).

### Grading the quality of evidence

The “Grades of Recommendations, Assessment, Development, and Evaluation” (GRADE) tool was used to determine the certainty of the evidence of the updated meta-analysis (36). The evidence for each included outcome was rated as having high-, moderate, low-, or very low-quality based on the study design, risk of bias, inconsistency, indirect evidence, imprecision, and publication bias. The overall score was downgraded by one score when ≤ 60% of the studies were at low risk of bias, as well as when inconsistency (*I^2^* >50%), indirect evidence and imprecision (wide CIs). The summary of Findings (SoF) table was created using GRADEpro GDT (37) (McMaster University, ON, Canada).

## Results

### Study selection

**Figure 1:**
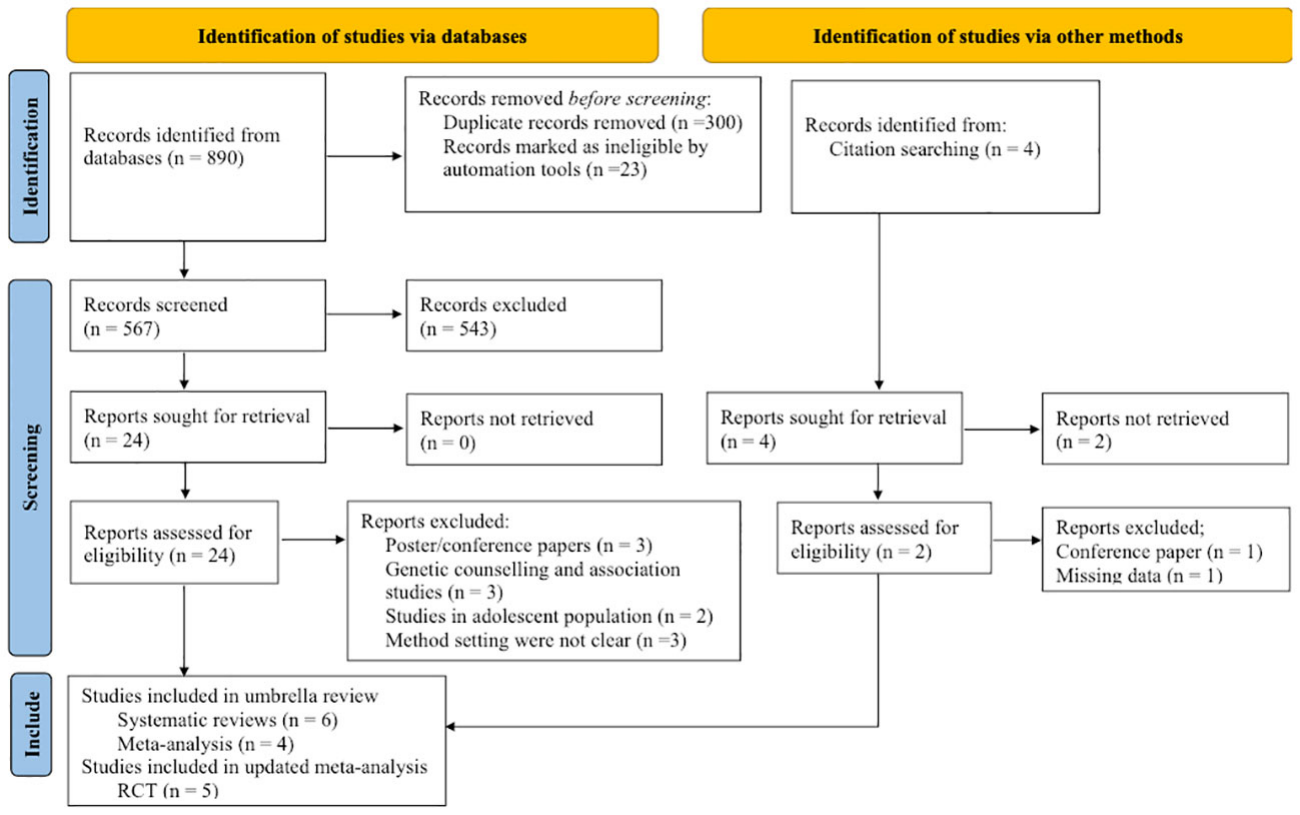
Flow diagram of the studies according to the Preferred Reporting Items for Systematic Reviews and Meta-Analyses (PRISMA).

The PRISMA flow diagram presents the study selection, inclusion process, and search results (**Figure 1**). A total of 890 studies (577 systematic reviews and meta-analyses and 313 RCTs) were identified through a systematic search on four selected databases. Additionally, four studies were retrieved through a manual search of references of all included studies. After removing duplications and title and abstract screenings, 28 studies were assessed in the full-text screening against the eligibility criteria. The excluded studies were poster or conference papers (five studies), studies in adolescence (two studies), genetic association and counselling studies (three studies) and other issues (three studies). In full-text screening, 13 studies were excluded, and the list of articles with their reasons for exclusion is available in the **Supplementary Table 1**. Finally, six meta-analyses and four systematic reviews were included for the umbrella review, and five studies were included for the updated meta-analysis. One eligible study (38) was excluded from the analysis due to missing data. The study conducted by McCarthy et al. (38), was a single-blind study aimed at examining the clinical utility of PGx testing in patients with bipolar disorder (BD), major depressive disorder (MDD), and post-traumatic stress disorder (PTSD). The study reported a significant benefit of PGx testing for PTSD patients but not for BD and MDD patients. However, this study was excluded from the analysis because it did not provide the number of MDD patients nor the events in the PGx and TAU groups.

### Characteristics of included studies

#### Systematic reviews and meta-analyses

The umbrella review comprised a total of six meta-analyses and four systematic reviews. A comprehensive description of all the included meta-analyses and systematic reviews can be found in **Table 1**. These studies included more than 17000 adult patients with depression. The majority (71%) of these patients were female, with ages ranging from 18 to 80 years old and an average age of 48 years. Over 90% of the participants were of Caucasian or non-Hispanic white ancestry. The remaining participants were Black (2%), Hispanic (1%), Asian (1%), Native American (0.1%), and other ethnicities (0.1%). All the meta-analyses evaluated the efficacy but not the safety of utilizing PGx testing in depression. The efficacy of the PGx-guided antidepressant medications was examined by measuring symptom improvement (22, 23), rate of remission (21, 22, 40, 43, 44), and rate of response (22, 40, 43). Two studies (22, 39) and one meta-analysis (23) were limited to primary studies that utilized particular genotyping platforms such as GeneSight Psychotropic test and Neuropharmagen, respectively. Rosenblat et al. (43), and Jokic et al. (45), also compared outcomes between different PGx testing panels such as GeneSight, Neuropharmagen, NeuroIDgenetix and CNSDose. In addition to evaluating the efficacy, systematic review studies analyzed the impact of PG-guided antidepressant treatment on the safety, tolerability, therapeutic decision patterns and satisfaction of patients with depression.

**Table 1:** Characteristics of systematic review and meta-analysis studies that are included in the umbrella review.

Reference	Review type	Characteristics of Included primary	Number of included studies/ participants	Outcomes	Main results
Health Quality Ontario (39)	Systematic review	Published before Feb 2016 and in English, Utilised GeneSight-guided medication	4/ 13,375	Response rate, remission rate, depression score, therapeutic decisions, patients' and clinicians' satisfaction	Improvements in response rate, measures of depression, and patients' and clinicians' satisfaction. No differences in remission rates.
Bousman et al., (21)	Meta-analysis	Randomised controlled trials (RCTs), adult participants (aged ≥18), Published before May 2018 and in English.	5/1737	Remission rates	Significant improvement (p **=** 0.005) in remission rates
Brown et al., (22)	Meta-analysis	Utilised the GeneSight Psychotropic test, Open-label and RCTs, including patients ≥18 years of age diagnosed with MDD	4/1556	Symptom improvement, Response rate and Remission rate	Significant increase in symptom improvements, remission and response rates
Ielmini et al., (40)	Systematic review and meta-analysis	RCTs, observational studies, or case-control studies, including patients aged ≥ 16 years, Published from 2000 to March 2021 in English	6/ 3,722‬	Remission and Response rates	Significant improvement of remission and response rates
Peterson et al., (41)	Systematic review	Published before Feb 2017 in English, Including adult patients	7/ 13,841‬	Remission rate, response rate and tolerability	CNSDose-guided medications significantly improve remission and reduced intolerability. Evidences are inconclusive about intolerability in ABCB1 and GeneSight based medications
Rosenblat et al., (42)	Systematic review	Published before Oct 2015 written in English, including adult patients (age 18-75 years), Open-label, non-randomised, nonblinded or lacking a control group	5/ 1,155	Symptom improvement, remission and response rates	- Four out of five studies reported significant improvement of depression symptom, response and remission rates
Rosenblat et al., (43)	Meta-analysis	Published before Dec 2017 written in English, Open-label, non-randomised or non-blinded studies with control group	6/ 1,329	Response and remission rates	Significant improvement of remission and response rates
Vilches et al., (23)	Meta-analysis	Analysis of three studies utilised Neuropharmagen-guided medications	3/450	Symptom improvement	Significant symptom improvement and the effect size is higher patients with moderate-severe depression
Brown et al., (44)	Meta-analysis	Prospective, controlled trial, published in English up to July 12th, 2022, Including adult participants (aged ≥18)	13/ 4,767	Remission rate	Significant (*P* = 0.001) remission rate
Jokic et al., (45)	Systematic review	Published in English before Jan 2020, randomised controlled trials, non-randomised studies, systematic reviews, and meta-analyses, including adult patients with major depression, Multi-gene pharmacogenomic testing intervention	14/ 3,497	Change in depression score, Response rate, Remission rate, intolerability to medication and Adverse events	No change in depression score, GeneSight– and NeuroIDgenetix–guided medication led to significant improvements in response and remission rates. CNSdose-guided medications led to significant improvement in remission rates. Genecept-guided medications led to no significant improvement in remission and response rates. Inconsistent impact of Neuropharmagen-guided meditations.

#### Randomized controlled trials

The study characteristics of the included RCTs are summarized in **Table 2**. Briefly, two RCTs (25, 46), one open-label study (47) and two *post hoc* analyses (5, 48) were included in the updated meta-analysis. A total of 3314 participants (47% female from baseline data) with moderate to severe depression were included in the five RCTs. The length of the follow-up period ranged from 8 to 24 weeks; however, the final outcomes were measured and analyzed at the end of the intervention (8 weeks). All the RCTs were preregistered at clinicaltrials.gov and included patients with a history of at least one previous inadequate treatment or intolerable side effect within the depressive episode. Patients were diagnosed with DSM-IV-TR-defined MDD, with MINI7.0 and SIGH‐D‐17 score >18 (25), self-rated and the site-rated 16-item Quick Inventory of Depression Symptomology (QIDS-SR16 and QIDSC16 scores ≥11 for diagnosis) (46, 48, 49) and Patient Health Questionnaire–9 (PHQ-9) score >9 (47). All studies used GeneSight-guided medications as an intervention, except for Perlis et al. (25),. Perlis et al. (25), used Genecept testing to genotype participants.

**Table 2:** Characteristics of RCTs studies that are included in the updated meta-analysis.

Study	Study design	Trial registration	Length(Weeks)	Inclusion	Exclusion	Outcome	Sample size(PGx/TAU)		Participants characteristics	PGx test (target genes)
							Baseline	Final Follow-up		
Tiwari et al., (46)	RCT (patient- and rater-blinded)	NCT02466477	52	18 years old, diagnosed with MDD, had inadequate response to at least one psychotropic medication	Patients were excluded if they had significant suicidal risk, severe co-occurring psychiatric or cognitive disorders, and/or unstable or significant medical conditions	Symptom improvement, Response at week 8, Remission at week 8	253/118	97/211	Mean age (40.7 years), Females (63.3%), Caucasian (83.6%), Asian (9.4%), Black (3%), Latin America (1.3%) and others (2.4%)	GeneSight® Psychotropic (CYP1A2, CYP2B6, CYP2C9, CYP2C19, CYP2D6, CYP3A4, HTR2A, and SLC6A4, MC4R, CNR1, NPY, GCG, HCRTR2, NDUFS1)
Perlis et al., (25)	RCT (patient- and rater-blinded)	NCT02634177	8	Age 18–75 years, with a primary diagnosis of nonpsychotic MDD, moderate to severe depression, failure of at least one prior adequate trial	severe personality disorder traits, all diagnosed personality disorders, diagnosed with bipolar and related disorders (lifetime diagnosis), trauma and stress‐related disorders, obsessive compulsive disorder and related disorders, Substance-related and addictive disorders, history of suicidal behaviour, four or more failed pharmacologic interventions, unstable or active medical condition	Symptom improvement, Response, Remission Safety and tolerability	151/153	146/150	Mean age (47.7 years), female (71.1%), Asian (03%), American Indian or Alaskan Native (1%), Black or African American (23.4%), Native Hawaiian or Other Pacific Islander (1%), White (72.7%) and other (1.6%)	Genecept Assay V 2.0 (45 variants of 7 pharmacokinetic cytochrome P450 genes and 12 variants of 11 pharmacodynamic or other genes)
Oslin et al., (47)	RCT (Raters blinded)	NCT03170362	24	Receiving care at VA medical centres, aged 18 to 80 years, with a diagnosis of MDD, History of at least 1 treatment episode	Active substance use disorder; bipolar illness; psychosis; borderline or antisocial personality disorder; treatment with an antipsychotic medication, methadone, buprenorphine, or naltrexone; augmentation treatment; and lack of a bank account for payments.	Remission, response and change in PHQ-9 score	966/976	826/842	Mean age (47.5), female (25.5%), African American/Black (18%), Asian Pacific Islander (3%), Native American/Alaskan (1%), White (68.5%), Other (9%) and Refused (1%)	GeneSight panel (CYP1A, CYP2B6, CYP2C19, CYP2C9, CYP3A4, CYP2D6, UGT1A4, UGT2B15, SLC6A4, HTR2A, HLA-B*1502, HLA-A*3101,
Forester et al., (48)	RCT (patient- and rater-blinded)	NCT02109939	24	Inadequate response to at least one medication, Post-hoc, 65 years of age or older at baseline	significant short-term suicide risk; bipolar disorder; current delirium or neurocognitive disorder; psychotic disorder or psychotic symptoms during the current or a previous depressive episode; a current substance use disorder; or a significant unstable medical condition.	Symptom improvement, Response and Remission at week 8	98/108	98/108	Average age (69.4 years), female (72.8%), White (91.3%), Black (7.8%), Asian (0.5%) and other (0.5%).	GeneSight Psychotropic (CYP1A2, CYP2C9, CYP2C19, CYP3A4, CYP2B6, CYP2D6 and HTR2A )
Thase et al., (49)	RCT (patient- and rater-blinded)	NCT02109939	24	Age 18 year or above diagnosed with MDD, Had at least one failed medication trial	Suicidal risk, some severe co-occurring psychiatric or cognitive disorders, and/or unstable or significant medical conditions	Symptom improvement, Response at week 8 and Remission at week 8	439/473	439/473	Average (48.7 years), female (70.8%) and Race: white (83.2%), Black (12.3%), Asian (2.4%), American Indian or Alaska Native (0.4%) and other (1.6%)	GeneSight Psychotropic (CYP1A2, CYP2C9, CYP2C19, CYP3A4, CYP2B6, CYP2D6, HTR2A, SLC6A4)

MDD, Major Depression Disorder; PGx, Pharmacogenomics; TAU, Treatment As Usual; RCTs, randomized controlled trials.

### Risk of bias and quality of the evidence

The methodological quality of the included systematic reviews and meta-analysis for the umbrella review and RCTs for the updated meta-analysis are summarized in the **Supplementary Tables 2**, **3**, respectively. According to the JBI-CAT, for systematic reviews and meta-analyses, four reviews were rated as high quality (21, 40, 43, 45), three reviews were rated as medium quality (39, 41, 44), and three (22, 23, 42) as low quality. All review protocols were preregistered in the PROSPERO database (21, 41, 44, 45). Bousman et al. (21), were sponsored by PGx testing providers, and most of the authors of two meta-analyses (22, 44) were employees or Consultants of PGx testing providers. Only two systematic reviews (39, 45) performed certainty of evidence and found that the quality of the included primary studies were from very low to moderate (GRADE).

For the five RCTs, according to the JBI-CAT checklists, three studies (46, 48, 49) scored 12 out of 13, one study (25) scored 11, and another study (47) scored 10. Moreover, all RCTs were fully or partially funded by industry, and performance bias was high because the physicians were unblinded. Patients were not blinded in one RCT (47), and two RCTs (5, 48) were *post hoc* analyses. Finally, the certainty of the evidence according to the GRADE approach was judged as low, mainly due to performance bias and inconsistency. The summary of findings for the GRADE assessment is available in the **Supplementary Table 4**.

### Symptom improvement

Symptom improvement, referred to as the depression score reduction, was measured by the mean percentage change in Hamilton Depression Rating Scale-17 (HDRS-17), Clinical Global Impression Scale (CGI) or Patient Health Questionnaire (PHQ). Three systematic reviews and two meta-analyses reported symptom improvement. Systematic review studies found inconsistent results for symptom improvement. Two studies (39, 42) found greater symptom improvement in PGx-guided treated patients. However, this did not reach significance in the RCTs. Jokic et al. (45), found no or little impact of PGx-guided medications in reducing depression scores compared to TAU. Jokic et al. (45), further evaluated the clinical significance of symptom improvement based on the predefined threshold, > 2 HAM-D17 scores mean change, as clinically meaningful and found only three out of the 14 studies with clinically relevant mean differences between the PGx-guided and TAU groups. Jokic et al. (45), also examined symptom improvement across different PGx testing panels and found inconsistent results within and across the tests. Overall, the quality of evidence of the primary studies included in this review was very uncertain, with GRADE from very low to low. Subgroup analysis showed symptom improvement was significantly larger among patients with baseline HAM-D17 ≥18, ≤ 5 years since diagnosis, previous failure of one to three antidepressants, and potential drug-drug interactions (45).

The two meta-analysis studies, Brown et al. (22), and Vilches et al. (23), found an additional 10.08% (95% CI: 1.67–18.50; p = 0.019) mean percentage and 0.34 (95% CI = 0.11–0.56, p-value = 0.004) standard mean reduction of depression (HAM-D17) scores, respectively, for patients who received PGx-guided medication compared with TAU. The improvement was more significant in open-label studies (Δ = 15.5, 95% CI: 8.72–22.29; p *<* 0.001) than in RCTs (Δ = 3.44%, 95% CI: 0.06–6.83; p = 0.046). Vilches et al. (23), reported higher symptom improvement (d**=** 0.42, 95% CI = 0.19–0.65, p-value = 0.004) for patients with moderate to severe depression.

**Figure 2:**
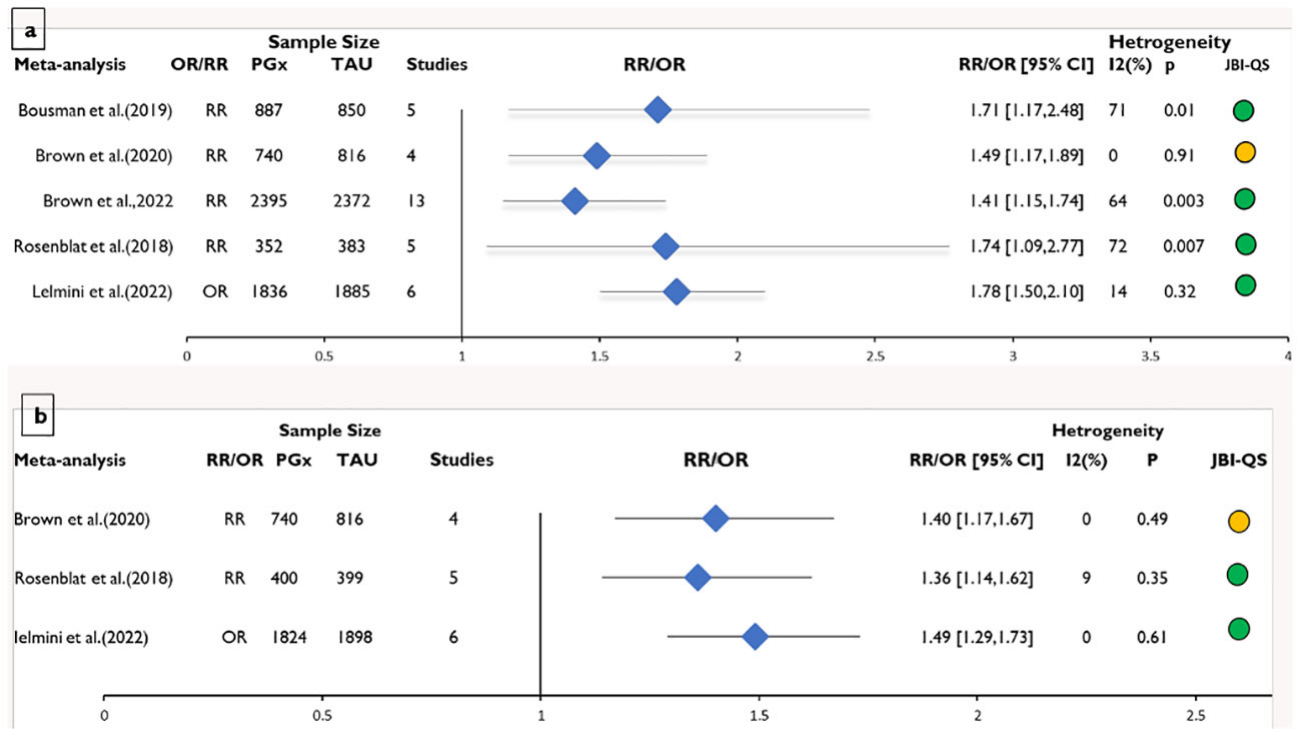
Meta-analysis of the risk ratios/odd ratios on the systematic reviews included in the umbrella review for the effect of pharmacogenomics (PGx)-guided medication vs. the treatment as usual (TAU) for **(A)** remission rate and **(B)** response rate. Effect sizes are expressed as RR (risk ratio) or OR (odd ratio). JBI-QS indicates critical appraisal score using the JBI tool; Green=9-11, yellow=5-8 and Red≤4.

Similarly, the random-effects model updated meta-analysis of five RCTs indicated symptom improvement was significantly higher (3.29%, 95% CI 0.6-5.98, p=0.02) in patients who received PGx-guided medication than in patients who received TAU (**Figure 2A**). The heterogeneity of the effects among the five RCTs was low and not significant (i^2^ = 20%, p=0.29). Symptom improvement was not significant when one of the three studies (46, 47, 49) was removed for sensitivity analysis (**Supplementary Table**-**5**). Symptom improvement was higher (3.95%, p=0.008) when Perlis et al. (25), was removed from the analysis. Subgroup analysis by tests utilized indicated symptom improvement was significant in GeneSight-guided treatment but not in Genecept-guided treatment when compared to TAU (3.95%, 95% CI = 1.64 - 6.25, p=0008).

### Rate of response

The rate of response was defined as a greater than 50% reduction in depression scores. Three meta-analyses (22, 40, 43) and four systematic reviews reported the rate of response of PGx-guided medication vs TAU. Systematic reviews found inconsistent results for the rate of response, and the quality of included primary studies was low to very low (GRADE). Systematic reviews of open-label non-randomized studies found that PGx-guided medication selection significantly improved the rate of response; however, it was lower in RCTs (41, 42). Jokic et al. (45), found a greater rate of response ranging from 25% to 74% in patients who received PGx-guided medication than TAU. However, the results were inconsistent across the PGx testing panels and depression scales. GeneSight– and NeuroIDgenetix–guided medications significantly improved the rate of response. No improvement for Genecept-guided medications and inconsistent results for Neuropharmagen-guided treatments. Greater improvement in response to PGx-guided antidepressant medications was noted in those patients who failed at least one previous antidepressant, who were < 60 years of age and had HAM-D17 ≥ 18 (45).

**Figure 3:**
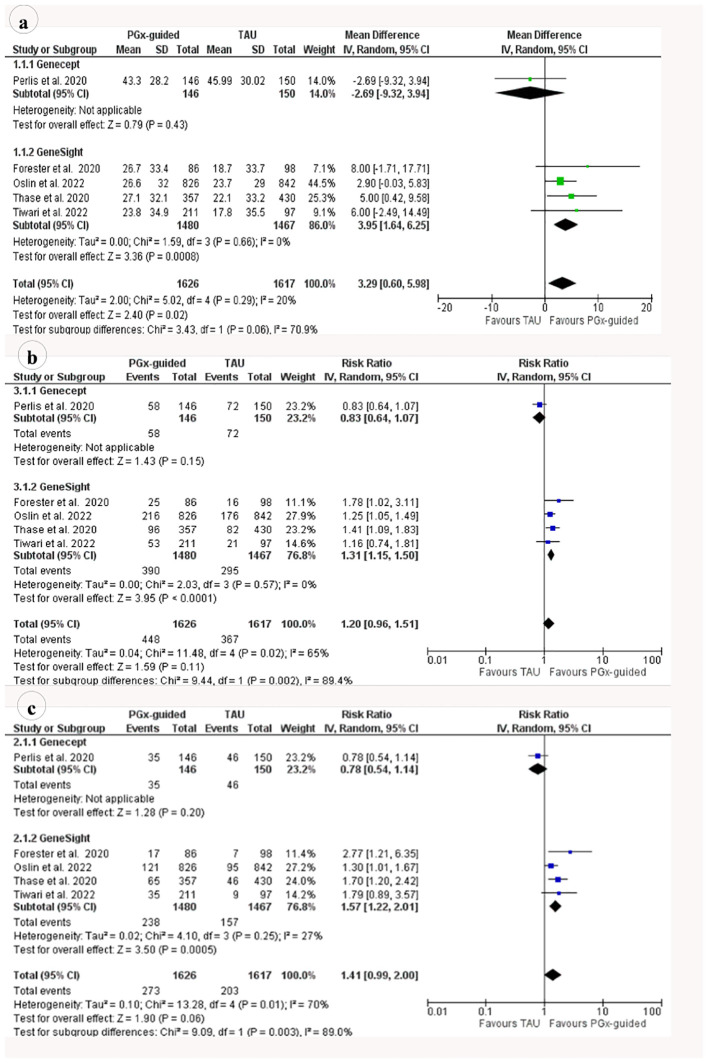
Meta-analysis of the risk ratios/odd ratios on the RCTs (randomised controlled trials) included in the updated meta-analysis for the effect of pharmacogenomics (PGx)-guided medications versus treatment as usual (TAU) for **(A)** mean differences in symptom improvement **(B)** Risk ratio for response **(C)** remission rates.

All the meta-analysis studies found significantly higher rate of response in patients who received PGx-guided medication than in patients who received TAU (**Figure 3A**). Individuals receiving PGx-guided treatment were between 36% to 49% more likely to respond to antidepressant medications than patients receiving TAU (**Figure 2A**). The rate of response was significantly higher in open-label studies than in RCTs (22, 43).

In contrast, the random-effect updated meta-analysis of RR from the five RCTs indicated no difference in response to medication among patients who received PGx-guided therapy vs TAU (RR=1.20, 95% CI:0.96-1.51, p=0.11) (**Figure 2B**). The significance of the result did not change when RCTs were removed from the analysis for sensitivity analysis, except for Perlis et al. (25), (**Supplementary Table**-**5**). The rate of response was 31% significantly (p < 0.0001) higher in the PGx-guided medication group when Perlis et al. (25), was removed from the analysis. Similarly, subgroup analysis based on the PGx tests showed a significantly higher rate of response in patients who received Genesight-guided medications (RR=1.31, 95% CI 1.15-1.5, p < 0.0001).

### Rate of remission

Remission was defined as HAMD-17 scores ≤ 7, QIDS-C16 scores ≤ 5, PHQ-9 scores ≤5 or HAM-D6 scores ≤ 4. Five meta-analyses and four systematic reviews reported the impact of PGx-guided medications on depression remission. The results from systematic reviews indicated that the impact of PGx-guided medication on depression remission was inconsistent and uncertain. Health Quality Ontario (39) found no difference between PGx-guided medication and TAU on depression remission. This review found that remission was significant when assessed using QIDS-C16, HAMD-17 or PHQ-9. Health Quality Ontario (39) included only studies that utilized GeneSight Psychotropic test. Peterson et al. (41), found a significant remission rate in studies that utilized ABCB1 and CNDose genotyping and inconsistent results in studies that utilized GeneSight genotyping. However, remission was defined as ≤ 10 HAMD-17 in the ABCB1 study and presents the possibility of overestimating effect size. Jokic et al. (45), found overall greater depression remission in patients who received PGx-guided treatment than TAU, ranging from 16.8% to 75% in PGx-guided treatments and 9% to 51.8% in TAU. However, the level of rate of remission was different across the PGx testing panels, and the quality of the included primary studies was Low–Very Low (GRADE). Overall, remission was significant in studies that utilized GeneSight, NeuroIDgenetix, and CNSDose tests but not significant in Neuropharmagen and Genecept tests (45).

All of the meta-analyses reported significant improvement in depression remission, and the effect size ranged from 1.41 to 1.78 (**Figure 3B**). Individuals receiving PGx-guided treatment were between 41% and 78% more likely to achieve remission than patients receiving TAU. Remission was significant in RCTs but not in open-label trials (22, 44).

In contrast, the rate of remission was not significant in the updated meta-analysis of five recent RCTs (RR=1.41, p=0.06) (**Figure 3C**). However, subgroup analysis based on the test indicated that Genesight-guided medication significantly improves the rate of remission (RR=1.57, 95% CI 1.22-2.01, p = 0.0005). The significance of the result did not change when RCTs were removed from the analysis for sensitivity analysis, except for Perlis et al. (25), (**Supplementary Table**-**5**). The rate of remission was 57% significantly (p < 0.0001) higher in the PGx-guided medication group when Perlis et al. (25), was removed from the analysis.

### Adverse effects, therapeutic decision, and satisfaction

Adverse effects were expressed as mean/proportion of adverse effects, intolerability rate and the Frequency, Intensity, and Burden of Side Effects Ratings (FIBSER) scale. Three systematic reviews reported adverse effects and intolerability, defined as the requirement to reduce the dose or stop medications. The quality of evidence was characterized as low (GRADE). Compared to TAU, Neuropharmagen- and CNDose-guided treatments significantly reduced adverse effects, the proportion of patients taking sick leave and intolerability to medications (39, 41, 45). Genecept- and GeneSight-guided medications have little or no effect on adverse effects (45). However, patients who switched to medications congruent to the GeneSight test at week 8 had significantly lower adverse effects than those who remained on incongruent medications (40). Ielmini et al. (40), found contradicting results regarding tolerability among patients who received PGx-guided treatment vs TAU but did not conduct separate analyses among the different testing panels.

Two systematic reviews (39, 45) reported the impact of PGx testing on the therapeutic decision, the proportion of individuals that switched, augmented, discontinued or adjusted their medication. Overall, these studies found inconsistent results on the impact of PGx testing on the therapeutic decision. The impact was significant in open-label studies or among those patients who received medications classified within the red bin (use with caution and more frequent monitoring) or yellow bin (use with caution). One systematic review (39) assessed the impact of PGx-guided medication on customer satisfaction and reported that GeneSight-guided care significantly improves patient and clinician satisfactions.

## Discussion

This study is the first to conduct an umbrella review and updated meta-analysis on the efficacy and safety of PGx-guided antidepressant prescribing in patients with depression. Based on the four systematic reviews, six meta-analyses and five RCTs, patients who receive PGx-guided treatment are more likely to respond to antidepressants with lower adverse effects compared to patients who receive TAU. Indeed, PGx-guided medications resulted in significantly better depression symptom improvement, response, and remission rates. However, our review also showed that the evidence on the impact of PGx testing in depression is highly variable, heavily influenced by study design and the type of PGx test. In addition, most of the studies focused on assessing the efficacy of PGx-guided medication. However, there is limited evidence on the safety of PGx-guided medications to treat depression. Finally, there is little or no evidence about the impact of PGx-guided antidepressant medication on suicide, relapse, recovery, or quality of life. After the completion of our study, three additional systematic reviews and meta-analysis studies (50–52) that evaluated the efficiency of PGx-guided antidepressant selection were published. However, these studies incorporated primary studies that are already included in this review.

The umbrella review and updated meta-analysis showed that PGx-guided medication was significantly associated with greater symptom improvement than TAU. However, the level of improvement was lower in the updated meta-analysis than in previous meta-analyses (22, 23). This fact could be partially explained by the pooling of results from studies that utilized different testing panels in the updated meta-analysis (22). In addition, our review showed that the type of study design influenced the effect size of the symptom improvement. In fact, symptom improvement due to PGx-guided medication was significantly higher in open-label studies than in RCTs (22, 39, 42). This could be explained by the placebo effect, i.e., participants’ awareness of the intervention and expectation of a better outcome (22, 41). Therefore, it seems that symptom improvement might not be a reliable outcome for evaluating the efficacy of PGx-guided antidepressant medication selections, even though it was the primary outcome in most studies. Furthermore, symptom improvement was expressed as the mean percentage of change, which is statistically inefficient and highly sensitive to changes in variance (53).

The umbrella review showed that the rate of remission and rate of response were significantly higher in patients who received PGx-guided antidepressant medication than in TAU. In contrast, the updated meta-analysis indicated no significant differences in the rate of response and rate of remission among the two treatment groups. However, the subgroup analyses by PGx test type showed significantly higher rate of response and rate of remission in patients who received GeneSight-guided medications than those who received TAU. This is in line with previous evidence and indicates that the effectiveness of PGx testing might depend on the type of testing panels utilized (22, 45). Overall, effect size estimates in the updated meta-analysis were smaller than those reported in previous meta-analyses (21–23, 44). The updated meta-analysis was expected to show higher benefit of PGx-guided medications because three out of five RCTs (25, 46, 47) included patients with moderate to severe depression and with a history of at least one previous inadequate treatment. In addition, one RCT (49) was *post hoc* analysis on a subset of patients with predicted drug-gene interactions. Previous reports state that PGx testing is more beneficial to patients with moderate–severe depression, previous history of inadequate drug response or predicted gene-drug interactions (12, 44). Unlike symptom improvement, the study design had a lesser influence on the rate of response and no influence on the rate of remission. Therefore, the rate of remission seems more stable and less susceptible to the placebo effect and represents a more clinically translatable efficacy measurement of PGx interventions in depression.

The benefits of PGx-guided antidepressant prescribing may depend on the type of PGx testing panel utilized. Here, the CNSDose-guided prescribing significantly improved the rate of remission and reduced adverse effects, and NeuroIDgenetix-guided prescribing improved both the rate of remission and the rate of response. Nevertheless, there were heterogeneous results for Neuropharmagen and Gensight tests, and no significant improvement was observed for the Genecept test. This inconsistency across the different PGx tests is intriguing and may have important implications for clinical practice.

One reason for the different clinical outcomes between PGx tests could be the tests’ proprietary or “black box” nature. The number of genes, their variants, and the algorithms used to predict phenotype differ between PGx tests (45). So do the prescribing recommendations, that can be proprietary or taken directly from various clinical implementation sources, including FDA recommendations, CPIC guidelines or the Dutch Pharmacogenomics Working Group (DPWG) guidelines (54). Bousman et al. (21), compared four PGx testing panels using the same five patients and reported disagreements in up to 67% of genotype results, 80% of phenotype results and 44% of antidepressant medication recommendations. In addition, Blazy et al. (11), reported up to 32% of phenotype assignment disagreement between testing panels and CPIC guidelines. Another reason for heterogeneity among testing panels could be the low quality of genotyping techniques. Almost all currently available PGx tests use array-based targeted genotyping (55, 56). However, these techniques cannot detect unknown, rare, and complex structural variants and hence can potentially miss clinically significant allelic variants and increase the possibility of false negatives (14). Recent studies indicated that up to 40% of the variations are attributed to rare variants (57–59). Therefore, it is crucial to consider both common and rare alleles to realize the full potential of PGx-guided pharmacotherapy. Overall, results are not translatable across testing panels. Therefore, evaluating and translating PGx-guided medication should be panel-specific (44, 45). This is completely impractical in clinical practice. The solution is to develop a standardized and straightforward PGx translation protocol monitored by external regulations (44). The heterogeneity among the testing panels is expected to reduce with the growing demands and efforts for standardization, such as Standardizing Laboratory Practices in Pharmacogenomics (STRIPE) (44).

### Limitations

Several shortcomings need to be considered while interpreting the results. First, the included RCTs have methodological limitations due to treating clinicians who were not blinded in all the RCTs and patients who were not blinded in one RCT (47). Therefore, these studies can have a placebo effect due to performance bias or the clinician’s expectations of success. In addition, studies relied on different depression scales and tests, which were pooled. Second, several primary studies were included across various reviews, indicating a high percentage of overlapping primary studies among the included systematic reviews and meta-analyses. Third, most participants were female Caucasians with moderate depression and most studies excluded patients with comorbid psychiatric conditions. This can limit the translatability of the results to broader real-world patient populations. Lastly, the included studies had high heterogeneity of results. Despite conducting subgroup analyses to address this issue and discussing possible sources of heterogeneity, these limitations could still influence the generalizability of the findings in clinical settings. Therefore, it is crucial to consider these limitations when interpreting and utilizing the current study’s findings.

## Conclusion

The successful clinical implementation of PGx-guided antidepressant prescribing depends on many factors, including analytical and clinical validity, prescriber and patient acceptance, and economic feasibility. This umbrella review and updated meta-analysis showed that PGx-guided antidepressant prescribing is associated with improved clinical outcomes in patients with depression compared to TAU. However, the benefits of this precision medicine approach may depend on the type of PGx test used to generate prescribing recommendations. The absence of standardization across PGx testing platforms, inaccurate genotyping, and low patient diversity in clinical studies (predominantly female Caucasians with moderate depression and few co-morbidities), remain areas of uncertainty about PGx testing and depression. Future RCTs should improve genotyping accuracy (long-read sequencing) to further understand the potential benefits of PGx-guided antidepressant prescribing. Furthermore, determining which antidepressants (drugs) benefited more from PGx utilization for improved response would be valuable input for the regulatory and consortia bodies for further investigation and confirmations.

## Data availability statement

The original contributions presented in the study are included in the article/**Supplementary material**. Further inquiries can be directed to the corresponding author.

## Author contributions

KT: Writing – review & editing, Writing – original draft. LZ: Writing – review & editing, Data curation. RF-R: Writing – review & editing, Methodology. DA: Writing – review & editing, Supervision. MM: Supervision, Writing – review & editing, Methodology. TP: Writing – review & editing. ML: Writing – review & editing, Supervision.
