# Supplementary material for: Efficacy and safety of pharmacogenomic-guided antidepressant prescribing in patients with depression: an umbrella review and updated meta-analysis
Source: Front Psychiatry. 2024 Jul 11;15:1276410. doi: 10.3389/fpsyt.2024.1276410 (PMC11289719; doi:10.3389/fpsyt.2024.1276410)
Supplement: Supplementary file 1 [file Table_1.docx]

**Supplementary materials:**

**Efficacy and safety of pharmacogenomic-guided antidepressant prescribing in patients with depression: An umbrella review and updated meta-analysis**

Kiflu G Tesfamicael^1,2^, Lijun Zhao^2,3^, Rubén Fernández-Rodríguez^4^, David L Adelson^1^, Michael Musker^5^, Thomas M Polasek^6^, Martin David Lewis^1,2, *^

*^1^School of Biological Sciences, University of Adelaide, Adelaide, SA, Australia. ^2^Lifelong Health, South Australian Health and Medical Research Institute (SAHMRI), Adelaide, SA, Australia. ^3^Adelaide Medical School, University of Adelaide, Adelaide, SA, Australia. ^4^Universidad de Castilla-La Mancha, Health and Social Research Center, Cuenca. Spain.^5^Clinical and Health Sciences, University of South Australia (UniSA), Adelaide, SA Australia, ^6^Centre for Medicine Use and Safety, Monash University, Melbourne, VIC, Australia. *Corresponding author martin.lewis@adelaide.edu.au*

**Index**

| ID | Subheading |
| --- | --- |
| Table S1 | List of excluded studies with the reason of exclusion |
| Table S2 | Quality assessment for the systematic review and meta-analysis according to the JBI tool |
| Table S3 | Quality assessment for the randomised controlled trials according to the JBI tool |
| Table S4 | Quality of evidence assessment according to GRADE approach for the randomised controlled trials for symptom improvement, response, and remission rates. |
| Table S5 | Sensitivity analyses for symptom improvement, response and remission rate |
| Table S6 | Logic-grid for meta-analyses and systematic review studies searches on PubMed, Ovid PsycINFO, Ovid Embase and Cochrane library databases |
| Table S7 | Logic-grid for Randomized Controlled Trials (RCTs) searches on PubMed, Ovid PsycINFO, Ovid Embase and Cochrane library databases. |

**Table S1**: **List of excluded studies with the reason of exclusion**

| Author (year) | Type of Study | Title | Reason of exclusion |
| --- | --- | --- | --- |
| Niistu et al. (2013) | Meta-analysis | Pharmacogenetics in major depression: A comprehensive meta-analysis | Genetic association study |
| Aboelbaha et al. (2021) | Systematic review | Effect of pharmacogenetic-based decision support tools in improving depression outcomes: A systematic review | Pooled data from selected meta-analyses studies and RCTs |
| Moldovan et al. (2017) | Meta-analysis | The Efficacy of Genetic Counselling for Psychiatric Disorders: a Meta-Analysis | Genetic Counselling |
| M Kata and Aserretti (2010) | Review and meta-analysis | Meta-analysis of antidepressant pharmacogenetic findings in major depressive disorder | Genetic association study |
| Arias A (2019) | Meta-analysis | A meta-analysis of combinatorial pharmacogenetic guided antidepressant treatment for major depressive disorder | Poster/conference paper |
| Bunka et al. (2021) | Systematic review and meta-analysis | Evaluating treatment outcome in pharmacogenomic-guided care for major depression; a rapid review and meta-analysis. | Poster/conference paper |
| Voort et al.,2022 | RCTs | A Randomized Controlled Trial of Combinatorial Pharmacogenetics Testing in Adolescent Depression | Study in Adolescent |
| Kennedy et al. (2020) | RCTs | Clinical utility of combinatorial pharmacogenetic testing in depression: Canadian patient- and rater-blinded, randomised, controlled trial | Poster/conference paper |
| Brow et al. (2020) | RCTs (*Post hoc*) | Combinatorial pharmacogenetic testing improves response and remission for patients over 65 with depression who have failed one medication trial | Poster/conference paper |
| Claudio-Campos et al. (2021) | Pragmatic stud | Acceptability, Feasibility, and Utility of Integrating Pharmacogenetic Testing into a Child Psychiatry Clinic | Study in Paediatric population |
| Wilhelm et al. (2021) | RCTs | Safety of using a combinatorial pharmacogenomic test for patients with major depressive disorder in the guided trial | Poster/conference paper |
| Papastergiou et al. (2021) | RCTs | Pharmacogenomics guided versus standard antidepressant treatment in a community pharmacy setting a Randomized controlled trial | Wrong setting (combined analysis for depression and generalised anxiety) |
| Ruaño et al. (2020) | RCTs | Results of the CYP-GUIDES randomised controlled trial: total cohort and primary endpoints | Wrong setting, the symptom improvement, response and remission rate were not analysed |

**Table S2: Quality assessment for the systematic review and meta-analysis according to the JBI tool.**

| Author | Q1 | Q2 | Q3 | Q4 | Q5 | Q6 | Q7 | Q8 | Q9 | Q10 | Q11 |
| --- | --- | --- | --- | --- | --- | --- | --- | --- | --- | --- | --- |
| Health Quality Ontario (2017) | Y | Y | Y | Y | Y | N | N | Y | N | N | Y |
| Health Quality Ontario (2021) | Y | Y | Y | Y | Y | N | Y | Y | Y | Y | Y |
| Bousman et al. (2019) | Y | Y | Y | Y | Y | Y | Y | Y | N | Y | Y |
| Vilches et al. (2019) | Y | NA | NA | NA | Y | NA | NA | Y | N | Y | Y |
| Brown et al. (2022) | Y | Y | Y | N | Y | Y | Y | Y | Y | Y | N |
| Brown et al. (2020) | Y | Y | N | N | Y | U | N | Y | N | N | Y |
| Ielmini et al. (2022) | Y | Y | Y | Y | Y | Y | Y | Y | N | Y | Y |
| Peterson et al. (2017) | Y | Y | Y | Y | Y | N | N | Y | N | N | Y |
| Rosenblat et al. (2017) | Y | Y | Y | Y | Y | U | N | NA | NA | N | Y |
| Rosenblat et al. (2018) | Y | Y | Y | Y | Y | Y | Y | Y | Y | N | Y |

Y=Yes, N = No, NA = Not Available, U = Unclear

Q1: Is the review question clearly and explicitly stated?

Q2: Were the inclusion criteria appropriate for the review question?

Q3: Was the search strategy appropriate?

Q4: Were the sources and resources used to search for studies adequate?

Q5: Were the criteria for appraising studies appropriate?

Q6: Was critical appraisal conducted by two or more reviewers independently?

Q7: Were there methods to minimise errors in data extraction?

Q8: Were the methods used to combine studies appropriate?

Q9: Was the likelihood of publication bias assessed?

Q10: Were recommendations for policy and/or practice supported by the reported data?

Q11: Were the specific directives for new research appropriate?

**Table S3: Quality assessment for the randomised controlled trials according to the JBI tool.**

| Studies | Q1 | Q2 | Q3 | Q4 | Q5 | Q6 | Q7 | Q8 | Q9 | Q10 | Q11 | Q12 | Q13 | Included |
| --- | --- | --- | --- | --- | --- | --- | --- | --- | --- | --- | --- | --- | --- | --- |
| Forester et al. (2020) | Y | Y | Y | Y | Y | Y | Y | Y | Y | Y | Y | Y | N | Y |
| Oslin et al. (2022) | Y | Y | Y | N | N | Y | Y | Y | N | Y | Y | Y | Y | Y |
| Perlis et al. (2020) | Y | Y | Y | Y | N | Y | Y | Y | U | Y | Y | Y | Y | Y |
| Thase et al. (2019) | Y | Y | Y | Y | N | Y | Y | Y | Y | Y | Y | Y | Y | Y |
| Tiwari et al. (2022) | Y | Y | Y | Y | N | Y | Y | Y | Y | Y | Y | Y | Y | Y |
|  |  |  |  |  |  |  |  |  |  |  |  |  |  |  |

Y=Yes, N = No, U = Unclear

Q1: Was true randomisation used for assignment of participants to treatment groups?

Q2: Was allocation to treatment groups concealed?

Q3: Were treatment groups similar at the baseline?

Q4: Were participants blind to treatment assignment?

Q5: Were those delivering treatment blind to treatment assignment?

Q6: Were outcomes assessors blind to treatment assignment?

Q7: Were treatment groups treated identically other than the intervention of interest?

Q8: Was follow up complete and if not, were differences between groups in terms of their follow up adequately described and analysed?

Q9: Were participants analysed in the groups to which they were randomised?

Q10: Were outcomes measured in the same way for treatment groups?

Q11: Were outcomes measured in a reliable way?

Q12: Was appropriate statistical analysis used?

Q13: Was the trial design appropriate, and any deviations from the standard RCT design (individual randomisation, parallel groups) accounted for in the conduct and analysis of the trial?

**Table S4:** **Quality of evidence assessment according to GRADE approach for the randomised controlled trials for symptom improvement, response, and remission rates.**

| **Certainty assessment** | | | | | | | **№ of patients** | | **Effect** | | **Certainty** | **Importance** |
| --- | --- | --- | --- | --- | --- | --- | --- | --- | --- | --- | --- | --- |
| **№ of studies** | **Study design** | **Risk of bias** | **Inconsistency** | **Indirectness** | **Imprecision** | **Other considerations** | **PGx-guided medication** | **TAU** | **Relative**  **(95% CI)** | **Absolute**  **(95% CI)** |  |  |
| **Symptom Improvement (follow-up: mean 8 weeks; assessed with: Mean percentage)** | | | | | | | | | | | | |
| 5 | randomised trials | serious^a^ | serious^b^ | not serious | not serious | none | 1626 | 1617 | - | MD **3.29 higher**  (0.6 higher to 5.98 higher) | ⨁⨁◯◯  Low |  |
| **Response rate (follow-up: mean 8 weeks)** | | | | | | | | | | | | |
| 5 | randomised trials | serious^a^ | serious^c^ | not serious | not serious | none | 448/1626 (27.6%) | 367/1617 (22.7%) | **RR 1.20**  (0.96 to 1.51) | **45 more per 1,000**  (from 9 fewer to 116 more) | ⨁⨁◯◯  Low |  |
| **Remission rate (follow-up: mean 8 weeks)** | | | | | | | | | | | | |
| 5 | randomised trials | serious^a^ | serious^d^ | not serious | not serious | none | 273/1626 (16.8%) | 203/1617 (12.6%) | **RR 1.41**  (0.99 to 2.00) | **51 more per 1,000**  (from 1 fewer to 126 more) | ⨁⨁◯◯  Low |  |
|  |  |  |  |  |  |  |  | 0.0% |  | **0 fewer per 1,000**  (from 0 fewer to 0 fewer) |  |  |

**CI:** confidence interval; **MD:** mean difference; **RR:** risk ratio

**Explanations**

a. Clinicians were blinded in all the studies, patients were not blinded in one study, one study was terminated before the proposed follow-up and two studies are post hoc

b. Heterogeneity was not significant; however, the effect size of this outcome was expressed as mean percentage change, which is statistically inefficient and can be a source of bias (Vickers 2001). The pooled effect size was variable during sensitivity analysis i.e. reanalysis by omitting individual included studies.

c. High heterogeneity of effect size among studies (i2=65%, p=0.02)

d. High heterogeneity of effect size among studies (i2=70%, p=0.01)

**Table S5: Sensitivity analyses for symptom improvement, response and remission rate.**

**Symptom Improvement**

| Omitted Study | Effect Size | 95% CI |
| --- | --- | --- |
| Forester et al.,2020 | 2.93 | [0.07, 5.78] |
| Oslin et al. 2022 | 3.63 | [-0.82, 8.08] |
| Perlis et al. 2020 | 3.95 | [1.64, 6.25] |
| Thase et al. 2020 | 2.75 | [-0.88, 6.38] |
| Tiwari et al. 2022 | 3.01 | [-0.16, 6.17] |

**Response rate**

| Omitted Study | Effect Size | 95% CI |
| --- | --- | --- |
| Forester et al.,2020 | 1.15 | [0.91, 1.45] |
| Oslin et al. 2022 | 1.2 | [0.86, 1.68] |
| Perlis et al. 2020 | 1.31 | [1.15, 1.50] |
| Thase et al. 2020 | 1.15 | [0.87, 1.53] |
| Tiwari et al. 2022 | 1.21 | [0.93, 1.59] |

**Remission rate**

| Omitted Study | Effect Size | 95% CI |
| --- | --- | --- |
| Forester et al.,2020 | 1.28 | [0.91, 1.82] |
| Oslin et al. 2022 | 1.5 | [0.88, 2.57] |
| Perlis et al. 2020 | 1.57 | [1.22, 2.01] |
| Thase et al. 2020 | 1.34 | [0.86, 2.09] |
| Tiwari et al. 2022 | 1.36 | [0.91, 2.02] |

**CI:** Confidence interval

**Table S6:** Logic-grid for meta-analyses and systematic review studies searches on PubMed, Ovid PsycINFO, Ovid Embase and Cochrane library databases.

**Table S6a:** Logic grid for PubMed

| Pharmacogenomics | Depression | Study type |
| --- | --- | --- |
| **“Genetic Testing” [mh] OR**  "Pharmacogenetics"[mh] OR  “Pharmacogenomic Testing” [mh] OR  pharmacogenetics-guided [tiab] OR  pharmacogenetic-guided [tiab] OR  Pharmacogenetic-Based[tiab] OR Pharmacogenetics-Based[tiab] OR  “Combinatorial pharmacogenomics” [tiab] or  pharmacogen*[tiab] OR “pharmacogenetics testing” [tiab] OR  “pharmacogenomics testing” [tiab] OR  “Genetic testing” [tiab] OR  “Informed prescribing”[tiab] OR  “cytochrome 450”[tiab] OR  CYP2D6[tiab] OR  CYP2C19[tiab] | **“**Mental Disorders” [mh] OR “Mood disorders”[mh] OR  **“depression”[mh] OR**  “Depressive disorder”[mh] OR  “Depressive disorder,  major”[mh] OR  Depression[tiab] OR  “Major depressive disorder”[tiab] OR  “Mental illness”[tiab] OR  “Mood disorder”[tiab] OR  Antidepressant[tiab] | “meta-analysis” [tiab] OR “meta-analyses”[tiab] OR "systematic review"[tiab] OR  "umbrella review"[tiab] |

**Table S6b:** Logic grid for Ovid PsycINFO

| Pharmacogenomics | Depression | Study type |
| --- | --- | --- |
| **Exp genetic testing OR**  pharmacogenetics-guided.ti,ab OR  pharmacogenetic-guided.ti,ab OR  Pharmacogenetic-Based. ti,ab OR  Pharmacogenetics-Based. ti,ab OR  Combinatorial pharmacogenomics. ti,ab OR  pharmacogen*. ti,ab OR  pharmacogenetic testing. ti,ab OR  pharmacogenomic testing. ti,ab OR  Genetic testing. ti,ab OR  Informed prescribing.mp OR  cytochrome 450. ti,ab OR  CYP2D6. ti,ab OR  CYP2C19. ti,ab | Exp Mental Disorders OR  **Major Depression.sh OR**  **depression.sh OR**  **Mental disorder.ti,ab OR**  **Mood disorder.ti,ab OR**  **Depression.ti,ab OR**  **Major Depression disorder.ti,ab OR**  **Antidepressant.ti,ab** | meta-analysis.ti,ab OR meta-analyses.ti,ab OR systematic review.ti,ab OR  umbrella review.ti,ab |

**Table S6c:** Logic grid Ovid Embase

| Pharmacogenomics | Depression | Outcome |
| --- | --- | --- |
| **Exp genetic screening OR**  Pharmacogenetics.sh OR  Pharmacogenomics.sh OR  pharmacogenetic testing.sh OR  cytochrome 450.sh OR  **cytochrome P450 2D6.sh OR**  **cytochrome P450 2C19.sh OR**  pharmacogen*.ti,ab OR  pharmacogenetic testing.ti,ab OR  pharmacogenomic testing.ti,ab OR  genetic testing.ti,ab OR  pharmacogenetics-guided.ti,ab OR  pharmacogenomics-guided.ti,ab OR pharmacogenetic-Based.ti,ab OR pharmacogenomics-Based.ti,ab OR  Informed prescribing.ti,ab OR  Combinatorial pharmacogenomic.ti,ab OR  cytochrome 450.ti,ab OR  CYP2D6.ti,ab OR  CYP2C19.ti,ab | Mental disease.sh OR  Depression.sh OR  Exp major depression OR  depression.sh OR  mental disorders.ti,ab OR  **Mood disorder.ti,ab OR**  depression.ti,ab OR  major depressive disorder.ti,ab OR  antidepressant.ti,ab | meta-analysis.ti,ab OR meta-analyses.ti,ab OR systematic review.ti,ab OR  umbrella review.ti,ab |

**Table S7:** Logic-grid for Randomised Controlled Trials (RCTs) searches on PubMed, Ovid PsycINFO, Ovid Embase and Cochrane library databases.

**Table S7a:** Logic grid for PubMed and Cochrane library database

| Pharmacogenomics | Depression | Systematic review |
| --- | --- | --- |
| **“Genetic Testing” [mh] OR**  "Pharmacogenetics"[mh] OR  “Pharmacogenomic Testing” [mh] OR  pharmacogenetics-guided [tiab] OR  pharmacogenetic-guided [tiab] OR  Pharmacogenetic-Based[tiab] OR Pharmacogenetics-Based[tiab] OR  “Combinatorial pharmacogenomics” [tiab] or  pharmacogen*[tiab] OR “pharmacogenetics testing” [tiab] OR  “pharmacogenomics testing” [tiab] OR  “Genetic testing” [tiab] OR  “Informed prescribing”[tiab] OR  “cytochrome 450”[tiab] OR  CYP2D6[tiab] OR  CYP2C19[tiab] | **“**Mental Disorders” [mh] OR “Mood disorders”[mh] OR  **“depression”[mh] OR**  “Depressive disorder”[mh] OR  “Depressive disorder,  major”[mh] OR  Depression[tiab] OR  “Major depressive disorder”[tiab] OR  “Mental illness”[tiab] OR  “Mood disorder”[tiab] OR  Antidepressant[tiab] | "Randomised Controlled Trials as Topic"[mh] OR “Randomized Controlled Trial” [tiab] OR  “Controlled Clinical Trial” [tiab] OR  “Clinical Trial” [tiab] |

**Table S7b:** Logic grid for Ovid PsycINFO database

| Pharmacogenomics | Depression | Outcome |
| --- | --- | --- |
| **Exp genetic testing OR**  pharmacogenetics-guided.ti,ab OR  pharmacogenetic-guided.ti,ab OR  Pharmacogenetic-Based. ti,ab OR  Pharmacogenetics-Based. ti,ab OR  Combinatorial pharmacogenomics. ti,ab OR  pharmacogen*. ti,ab OR  pharmacogenetic testing. ti,ab OR  pharmacogenomic testing. ti,ab OR  Genetic testing. ti,ab OR  Informed prescribing.mp OR  cytochrome 450. ti,ab OR  CYP2D6. ti,ab OR  CYP2C19. ti,ab | Exp Mental Disorders OR  **Major Depression.sh OR**  **depression.sh OR**  **Mental disorder.ti,ab OR**  **Mood disorder.ti,ab OR**  **Depression.**ti,ab **OR**  **Major Depression disorder.**ti,ab **OR**  **Antidepressant.ti,ab** | **Exp Randomized Controlled Trials OR**  **Exp Clinical Trials OR**  **Randomized Controlled Trials**.ti,ab OR  **Clinical Trials.ti,ab OR**  Controlled Clinical Trial.ti,ab |

**Table S7c:** Logic grid for Ovid Embase database

| Pharmacogenomics | Depression | Outcome |
| --- | --- | --- |
| **Exp genetic screening OR**  Pharmacogenetics.sh OR  Pharmacogenomics.sh OR  pharmacogenetic testing.sh OR  cytochrome 450.sh OR  **cytochrome P450 2D6.sh OR**  **cytochrome P450 2C19.sh OR**  pharmacogen*.ti,ab OR  pharmacogenetic testing.ti,ab OR  pharmacogenomic testing.ti,ab OR  genetic testing.ti,ab OR  pharmacogenetics-guided.ti,ab OR  pharmacogenomics-guided.ti,ab OR pharmacogenetic-Based.ti,ab OR pharmacogenomics-Based.ti,ab OR  Informed prescribing.ti,ab OR  Combinatorial pharmacogenomic.ti,ab OR  cytochrome 450.ti,ab OR  CYP2D6.ti,ab OR  CYP2C19.ti,ab | Mental disease.sh OR  Depression.sh OR  Exp major depression OR  depression.sh OR  mental disorders.ti,ab OR  **Mood disorder.ti,ab OR**  depression.ti,ab OR  major depressive disorder.ti,ab OR  antidepressant.ti,ab | **Exp randomised controlled trial OR**  **Exp controlled clinical trial OR**  **clinical trial.sh OR**  **randomised controlled trial**.ti,ab OR  **controlled clinical trial.ti,ab OR**  clinical trial.ti,ab |
